# Understanding individual health-related social needs in the context of area-level social determinants of health: The case for granularity

**DOI:** 10.1017/cts.2024.519

**Published:** 2024-04-16

**Authors:** Andrew Telzak, Samantha Levano, Jessica Haughton, Earle C. Chambers, Kevin P. Fiori

**Affiliations:** 1 Department of Family and Social Medicine, Albert Einstein College of Medicine, Bronx, NY, USA; 2 Department of Pediatrics, Albert Einstein College of Medicine, Bronx, NY, USA; 3 Department of Epidemiology and Population Health, Albert Einstein College of Medicine, Bronx, NY, USA; 4 Department of Psychiatry and Behavioral Sciences, Albert Einstein College of Medicine, Bronx, NY, USA; 5 Office of Community and Population Health, Montefiore Health System, Bronx, NY, USA

**Keywords:** Health related social needs, social determinants of health, community-level social risk, geospatial mapping, ecological fallacy

## Abstract

**Introduction:**

Screening for health-related social needs (HRSNs) within health systems is a widely accepted recommendation, however challenging to implement. Aggregate area-level metrics of social determinants of health (SDoH) are easily accessible and have been used as proxies in the interim. However, gaps remain in our understanding of the relationships between these measurement methodologies. This study assesses the relationships between three area-level SDoH measures, Area Deprivation Index (ADI), Social Deprivation Index (SDI) and Social Vulnerability Index (SVI), and individual HRSNs among patients within one large urban health system.

**Methods:**

Patients screened for HRSNs between 2018 and 2019 (*N* = 45,312) were included in the analysis. Multivariable logistic regression models assessed the association between area-level SDoH scores and individual HRSNs. Bivariate choropleth maps displayed the intersection of area-level SDoH and individual HRSNs, and the sensitivity, specificity, and positive and negative predictive values of the three area-level metrics were assessed in relation to individual HRSNs.

**Results:**

The SDI and SVI were significantly associated with HRSNs in areas with high SDoH scores, with strong specificity and positive predictive values (∼83% and ∼78%) but poor sensitivity and negative predictive values (∼54% and 62%). The strength of these associations and predictive values was poor in areas with low SDoH scores.

**Conclusions:**

While limitations exist in utilizing area-level SDoH metrics as proxies for individual social risk, understanding where and how these data can be useful in combination is critical both for meeting the immediate needs of individuals and for strengthening the advocacy platform needed for resource allocation across communities.

## Introduction

Since the pioneering work of Engles and Virchow [1] in the mid-19^th^ century, the health of individuals and communities has been understood to be in large part socially determined. By the late 19^th^ century in the United States, W.E.B Du Bois had called attention to the ways that different social and environmental conditions impacted tuberculosis outcomes differently for blacks than for whites [2]. The World Health Organization began emphasizing the need to address the social causes of health in their landmark Alma-Ata Declaration on Primary Care in 1978 [3], and has since built on this to define the Social Determinants of Health (SDoH) as “the conditions in which people are born, grow, work, live, and age, and the wider set of forces and systems shaping the conditions of daily life” [4]. A robust infrastructure to measure and understand the SDoH has grown from these pioneering efforts, successfully making the case that the SDoH plays a large role in determining illness and health [5–8].

Under the umbrella term of “Social Determinants of Health,” specific nomenclature allows for further distinctions in our understanding of how health is socially determined. Individual *social causes of health*, such as food availability and housing quality, can act as either social assets or social risks for individuals, depending on the circumstances [9]. Social risk factors are defined specifically as adverse, measurable, individual-level social determinants of health [10]. Within this framework, health-related social needs (HRSNs) are self-reported individual needs that center individual preferences in the prioritization of social care at a particular moment in time [11].

Over the past decade, there has been a burgeoning body of literature exploring the links between SDoH, medical morbidity, and a variety of health outcomes [12–14]. This has led to several professional organization guidelines recommending the screening for HRSNs [15–18], and federal and state agencies proposing funding mechanisms to incentivize and reimburse for these activities [19–21]. However, screening for HRSNs can be challenging to implement and time-consuming for providers [22]. Given the challenges of collecting individual-level HRSN data, many have begun utilizing large, publicly available data sets to estimate the SDoH by geographic area [14,23]. A variety of aggregate measures are now available [24] which have been used as proxy measures for individual-level risk [25], at times with interventions designed to target individuals within these communities of “higher risk” [26]. At the same time, others have cautioned against this approach, highlighting not only how the various composite measures have different meanings in different contexts [24], but also the potential for harm and susceptibility to the ecological fallacy [27].

Comparisons between individual-level social risks and area-level SDoH metrics in a variety of settings have shown the limitations of this approach [28–33], finding that area-level indices are variable predictors of individual-level social risk. However, past studies have been conducted in geographically dispersed communities and among heterogeneous patient populations, drawing conclusions across a wide range of settings. Important gaps remain in our understanding of these relationships, in particular within historically marginalized communities that have been labeled as vulnerable en mass, without an understanding of the nuances of resiliency or access to resources. In a setting like the Bronx, NY, the narrative of population-wide poor health outcomes is defined by decades of divestment and marginalization that are easily identified with area-level metrics. Analyzing the data from a single, large urban health system’s HRSNs screening program may provide additional insight into the relationships between individual social risks and area-level SDoH metrics to better design multi-sectoral interventions that are needed to address immediate patient needs as well as target structural inequities.

The aim of this study was to assess how three area-level SDoH indices (the Area Deprivation Index [ADI] [34], Social Deprivation Index [SDI] [23], and Social Vulnerability Index [SVI] [35]) were associated with individual HRSN screening results among a sample of patients within one urban health system. Additionally, this study aims to visually display these findings geographically across the catchment area of the health system. These data together are important and complementary in how they can be used in actionable ways in clinical settings.

## Materials & methods

### Setting

This study was conducted in an urban, hospital-based primary care network in the Bronx, NY, and includes pediatric, internal medicine, and family medicine practices, with 10 designated Federally Qualified Health Centers. Since 2017, the health system has implemented a system-wide HRSN screening program [36].

### Study sample

**Figure 1.**
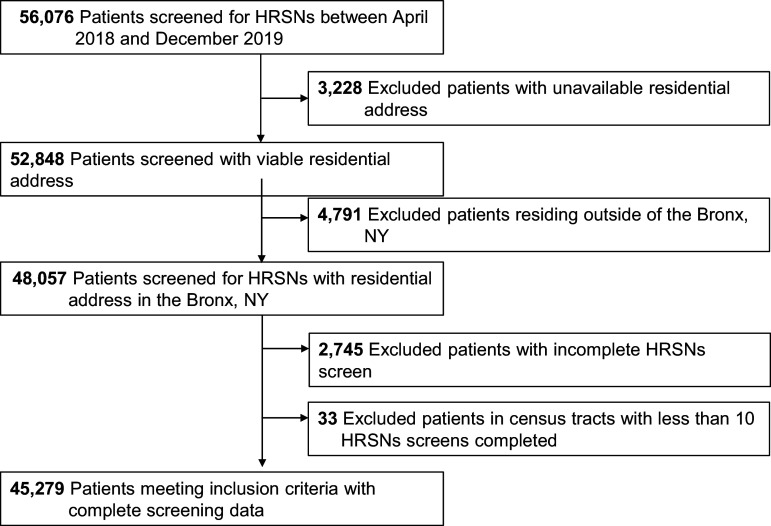
Derivation of study sample. HRSN = health-related social needs.

Patients (*N* = 56,076) were screened for HRSNs in the ambulatory care network between April 2018 and December 2019. Patients were excluded from the analysis if their residential address, and therefore census tract geographic identifier (GEOID), was unavailable (*N* = 3,228), or if they resided outside of the Bronx, NY (*N* = 4,791). The remaining patients (*N* = 48,057) were geocoded to a census tract. From this sample, patients were excluded from the analysis if HRSN screening data was incomplete (*N* = 2,745), or if there were fewer than 10 HRSN screens completed in the assigned census tract (*N* = 33), for a total sample of 45,279 individuals (Fig. 1).

### Measures

#### Individual-level HRSNs

The health system adapted a standardized 10-item HRSN screening tool from a widely used instrument, the Health Leads screening toolkit [37], after an extensive pilot process involving key stakeholders. The tool was integrated into the health system’s Electronic Health Record (EHR), Epic, and self-administered in patients’ preferred language in a pragmatic fashion. While not every patient in the health system was screened for HRSN within the study period, each clinical team was given the discretion to decide which patients to screen (i.e. new patients, patients seen for annual physicals, patients with high-risk comorbidities) [38]. The primary outcome for this analysis was a binary variable defined as the presence of at least one identified HRSN. HRSNs were defined by the following categories of need: housing quality, housing instability, food insecurity, health-related transportation, healthcare costs, utility costs, domestic disputes, child or adult care, legal help, and interpersonal violence (Supplemental Table A).

### Patient characteristics

Additional demographic characteristics were collected from the EHR for each patient screened. These characteristics included age (continuous), sex (categorized: male, female), preferred language (categorized: English, Spanish, other, missing indicator), and health insurance at the screening visit (categorized: Medicaid, Medicare, commercial, uninsured). Race and ethnicity (categorized: Non-Hispanic Black, Non-Hispanic White, Hispanic, missing indicator) were also collected from self-identified data in the EHR and used here as a proxy for unmeasured confounding that data from the EHR are not designed to collect.

### Area-level SDoH

To compare area-level SDoH metrics and individual-level social risks, we selected three frequently used area-level measures: ADI, SDI, and SVI. Each of these indices included slightly different variables and are all used frequently to understand area-level SDoH (see Supplemental Table B for comparison of variables) [39–41].

The ADI is a composite, factor-based index that utilizes the American Community Survey (ACS) Five Year Estimates to rank census block groups by 17 socioeconomic indicators, including measures on income, education, employment, and housing quality [34]. The ADI is constructed by region of interest, which allows for comparison at both the state and national levels. The census block group is the geographic unit of construction for the ADI, so block groups were converted to census tracts to utilize the ADI mean rank (as has been done previously [42]) and to compare with the SDI and SVI census tracts. When there were multiple census block groups per census tract, a mean value was created of census block group values (which accounts for some differences in the total N of the sample and analyses including ADI scores). Higher ADI rankings are indicative of a greater likelihood of adverse SDoH, with a potential range of scores between 0 and 10.

The SDI is constructed based on seven census tract-level characteristics collected in the ACS Five Year Estimates. These characteristics include: percent living in poverty, percent with less than 12 years of education, percent single parent household, percent living in rented housing unit, percent living in overcrowded housing unit, percent of households without a car, and percent unemployed adults under 65 years of age. Higher SDI scores represent greater likelihood of adverse SDoH, with a score of 75, for example, considered to have a greater likelihood of adverse SDoH than 75% of census tracts nationally [23]. The potential scores range between 0 and 100.

The SVI was developed by the Agency for Toxic Substances and Disease Registry of the Centers for Disease Control and Prevention to identify communities’ susceptibility to hazardous events on health [35]. The SVI determines social risk at the census tract level based on 15 social factors collected by the ACS. The SVI variables are grouped into four related themes: socioeconomic status, household composition and disability, minority status and language, and housing type and transportation. Census tracts are assigned an overall ranking with comparisons at the state and national levels. The overall ranking represents the proportion of census tracts that are equal to or lower than the tract of interest in terms of social vulnerability. Higher ranking indicates greater likelihood of adverse SDoH, with a potential range of scores between 0 and 1.

### Analytic approach

#### Area-level SDoH

Patient addresses were extracted from each individual patient health record and geocoded to census blocks through GEOID using the New York State Street and Address Composite geocoding services tool [43]. Census blocks were then converted to census tracts for this analysis. Although imperfect and certainly with limitations [44], census tracts were utilized here as proxy measures for “neighborhoods.” Area-level SDoH scores were categorized into tertiles for ease of interpretation, given the skewed distribution towards higher scores in the geographic area of interest (see supplemental figure A for histogram distributions of each score). Given that two of the three indices (ADI and SVI) utilized rank-based outcomes, this categorization allowed for understanding the variability at the extremes, which has been a noted limitation with area-level indices that are rank-based [24]. Tertiles for each index were categorized based on increasing SDoH risk (“low,” “medium” and “high” risk), with the reference group being census tracts at the lowest level of SDoH risk.

#### Patient characteristics, HRSNs, and area-level SDoH

Descriptive analyses were performed to assess the bivariate associations between patient characteristics and presence of HRSNs. Tertiles of each of the area-level SDoH indices were created to assess the association between these (low-risk, medium-risk, and high-risk) and individual social risks (presence or absence of HRSNs). Multivariable logistic regression models were then estimated to assess the association between area-level SDoH tertiles and presence of identified HRSNs. Covariates adjusted for in our model were selected based on previous literature suggesting their association with HRSNs, including age, sex, race and ethnicity, preferred language, and insurance payer. Model fit was also adjusted to account for clustering at the census tract level. Multivariable models were assessed for multicollinearity with Variance Inflation Factor (VIF) and Confidence (1/VIF). All p-values less than 0.05 were considered statistically significant. Statistical analyses were performed using STATA version 16.0 (StataCorp, College Station, Tx). All research was approved by the Albert Einstein College of Medicine Institutional Review Board. All social needs data were extracted from the EHR using Microsoft SQL Server, version 18, to query data from the Epic Electronic Health Record Data Warehouse.

#### Mapping

**Figure 2.**
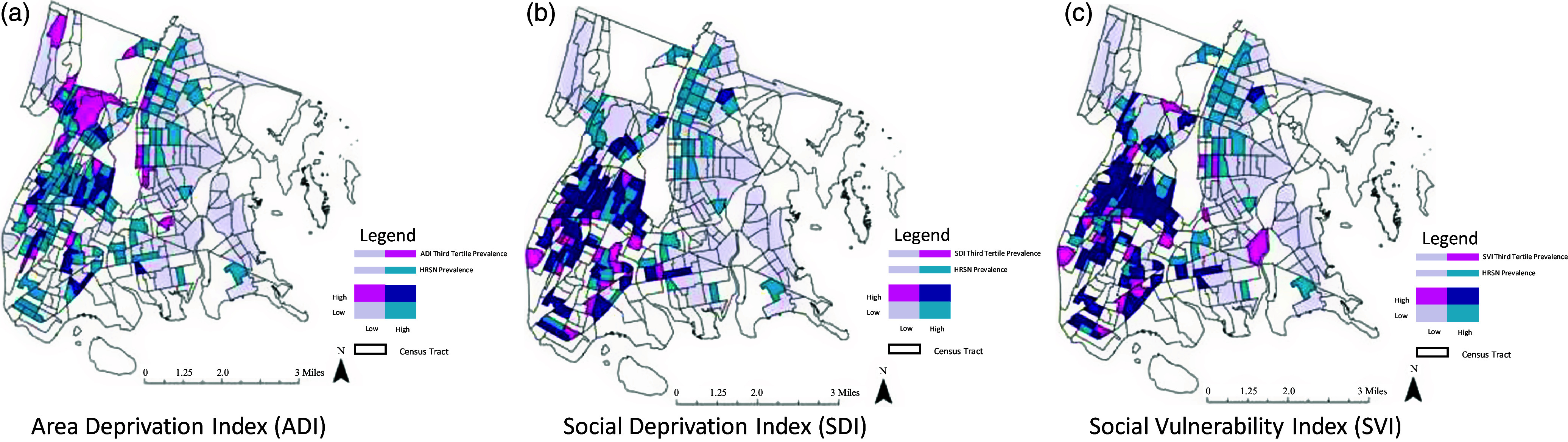
Choropleth maps.

We determined the count of patients per census tract with at least one HRSN and divided this measure by the total count of patients screened for HRSN per census tract to generate the HRSN prevalence within each census tract. Each census tract was then categorized into either *low individual-level HRSN* (less than the mean HRSN prevalence of 19.3%) or *high individual-level HRSN* (greater than or equal to the mean social need prevalence of 19.3%). Tertiles for each area-level SDoH measure were used to categorize each census tract as *low area-level SDoH risk* (tertiles 1 and 2) or *high area-level SDoH risk* (tertile 3). The binary *individual-level HRSN* variable within each census tract was then compared with the binary variables of area-level SDoH (for ADI, SDI, and SVI) through the creation of bivariate choropleth maps using ArcGIS Pro (version 3.1, Esri Inc., Redlands, CA). The final bivariate choropleth maps (Fig. 2) display the intersection of these two binary variables to visualize the spatial relationship between individual-level social risk and area-level SDoH measures in the study sample.

The color assignment is standardized across each map; however, the quantile distribution of each area-level measure changes according to the distribution of each area-level score. Individual census tracts with fewer than 10 patients screened for HRSNs during the study period are represented in white (and excluded from analysis, as previously described).

To understand the potential “predictive value” of area-level SDoH (i.e. how well these measures align with individually identified HRSN), the sensitivity and specificity of the area-level SDoH indices were calculated using count variables of the number of census tracts attributed to each combination of area-level SDoH and individual-level social risk (Supplemental Table D). Individual HRSN screening results were considered the true positive. Similarly, Positive Predictive Values and Negative Predictive Values of each area-level SDoH index were also calculated.

## Results

### HRSNs and patient characteristics

Between April 2018 and December 2019, 45,279 patients were screened for HRSNs in the Bronx, NY and included in this analysis (Table 1). The median age at screening was 33.5 years, with 60% identifying as female. 39.5% of patients identified as Hispanic, followed by 28.1% non-Hispanic Black. Race and ethnicity data was missing for 26.8% of patients. A majority of patients (79.5%) indicated their preferred language as English, with an additional 15.8% preferring Spanish. Almost half of patients were enrolled in Medicaid (45.1%) with the remaining payer mix consisting of commercial insurance (31.2%) or Medicare (18.5%).

**Table 1.** Patient characteristics

	Total, *N* (%)	Zero HRSNs (*N*, %*)	One or More HRSNs (*N*, %*)
**Total**	45,279	36,883 (81.5)	8,396 (18.5)
**Age (median [IQR])**	33.5 [11.4–58.8]	33.2 [11.4–59.4]	34.4 [11.1–56.8]
**Sex**			
Female	27,018 (59.7)	21,922 (59.4)	5,096 (60.7)
Male	18,261 (40.3)	14,961 (40.6)	3,300 (39.3)
**Race and ethnicity**			
Non-Hispanic Black	12,737 (28.1)	10,510 (28.5)	2,227 (26.5)
Non-Hispanic White	1,518 (3.4)	1,293 (3.5)	225 (2.7)
Hispanic	17,866 (39.5)	14,105 (38.2)	3,761 (44.8)
Other	1,005 (2.2)	883 (2.4)	122 (1.5)
Missing	12,153 (26.8)	10,092 (27.4)	2,061 (24.6)
**Preferred Language**			
English	35,995 (79.5)	29,528 (80.1)	6,466 (77.0)
Spanish	7,157 (15.8)	5,506 (14.9)	1,651 (19.7)
Other	1,139 (2.5)	991 (2.7)	148 (1.8)
Missing	^a^989 (2.2)	858 (2.3)	131 (2.2)
**Payer**			
Commercial	14,110 (31.2)	12,570 (34.1)	1,540 (18.3)
Medicaid	20,438 (45.1)	15,648 (42.4)	4,790 (57.1)
Medicare	8,357 (18.5)	6,808 (18.5)	1,549 (18.5)
Uninsured	2,374 (5.2)	1,857 (5.0)	517 (6.2)

HRSN = health-related social needs, IQR = interquartile range.

* column percentages displayed.

Of the patients in the study sample, 18.5% reported one or more HRSN. Those with identified HRSNs were similar in age (34.4 vs 33.3 years) to those without HRSNs, but more likely to identify as Hispanic (44.8% vs 38.2%), with a slightly greater likelihood of Spanish as their preferred language (19.7% vs 14.9%). Individuals with identified HRSNs were also more likely to have Medicaid insurance (57.1% vs 42.4%) and much less likely to have commercial insurance (18.3% vs 34.1%) than those without HRSNs.

Of the 18.5% of patients who reported one or more HRSNs, housing quality (5.9%), food insecurity (5.8%), and healthcare transportation (4.6%) were the most commonly identified HRSNs (Supplemental Table C).

### HRSNs and area-level SDoH tertiles

We assessed the relationship between tertiles of each area-level SDoH measure and the presence of individual HRSNs (Table 2). ADI scores showed a greater percentage of individuals with identified HRSNs in the medium SDoH risk group (21.0%) than in either the low SDoH risk group (17.6%) or the high SDoH risk group (16.0%). Using the SDI score, we observed a greater percentage of individuals with HRSNs with greater area-level SDoH (high-risk > medium-risk > low-risk). For example, 12.4% of those living in geographic areas within the *low* SDI SDoH risk group identified HRSNs, as compared to 19.8% in the medium SDoH risk group and 23.2% in the high SDoH risk group. SVI trends appeared similar to the SDI trends noted above.

**Table 2.** Relationship of area-level social determinants of health tertiles with *individual-level health-related social needs*

	Total, *N*	Zero HRSNs, *N* (%*)	One or More HRSNs, *N* (%*)
**Area Deprivation Index (ADI)**			
Low SDoH Risk (Tertile 1)	14,537	11,974 (82.4)	2,563 (17.6)
Medium SDoH Risk (Tertile 2)	17,806	14,043 (78.9)	3,763 (21.1)
High SDoH Risk (Tertile 3)	12,923	10,858 (84.0)	2,065 (16.0)
**Social Deprivation Index (SDI)**			
Low SDoH Risk (Tertile 1)	13,979	12,248 (87.6)	1,731 (12.4)
Medium SDoH Risk (Tertile 2)	17,449	13,998 (80.2)	3,451 (19.8)
High SDoH Risk (Tertile 3)	13,851	10,637 (76.8)	3,214 (23.2)
**Social Vulnerability Index (SVI)**			
Low SDoH Risk (Tertile 1)	13,978	12,230 (87.5)	1,748 (12.5)
Medium SDoH Risk (Tertile 2)	15,258	12,242 (80.2)	3,016 (19.8)
High SDoH Risk (Tertile 3)	16,043	12,411 (77.4)	3,632 (22.6)

HRSN = health-related social needs, SDoH = social determinants of health.

* row-percentages displayed.

Table 3 shows the three separate multivariable logistic regression models, each predicting the odds of the presence of one or more HRSNs. For the ADI model, when adjusting for covariates, 15% greater odds of HRSNS was seen among those residing in the medium SDoH risk census tracts as compared to low SDoH risk census tracts (95% CI 1.06–1.25). A slightly lower odds of HRSNs was seen among those residing in high SDoH risk census tracts (as compared to low SDoH risk); however, this difference was not found to be statistically significant. SDI, as well as SVI indices revealed greater odds of the presence of HRSNs in medium and high SDoH risk census tracts, as compared to low SDoH risk census tracts, when adjusted for all covariates. For example, those individuals residing in medium SDI risk census tracts had 55% greater odds of reporting one or more HRSN than those in low SDI risk census tracts (95% CI 1.34–1.79). High SDI risk census tracts had 80% greater odds of reporting one or more HRSN than those in low SDI risk census tracts (95% CI 1.56–2.07). These trends appeared similarly for SVI scores.

**Table 3.** Multivariable logistic regressions of area-level social determinants of health on individual health-related social needs

	*Odds Ratios [95% Confidence Intervals]*		
	Area Deprivation Index (ADI)	Social Deprivation Index (SDI)	Social Vulnerability Index (SVI)
Low SDoH Risk (Tertile 1)	[REF]	[REF]	[REF]
Medium SDoH Risk (Tertile 2)	1.15 [1.06–1.25]	1.55 [1.34–1.79]	1.53 [1.32–1.77]
High SDoH Risk (Tertile 3)	0.90 [0.73–1.10]	1.80 [1.56–2.07]	1.74 [1.50–2.01]
**Age (median [IQR])**	1.00 [0.99–1.00]	1.00 [0.99–1.00]	1.00 [0.99–1.00]
**Sex**			
Male	[REF]	[REF]	[REF]
Female	1.04 [0.99–1.10]	1.04 [0.98–1.10]	1.04 [0.98–1.10]
**Race and ethnicity**			
Non-Hispanic Black	[REF]	[REF]	[REF]
Non-Hispanic White	0.83 [0.70–0.98]	0.94 [0.80–1.11]	0.94 [0.80–1.10]
Hispanic	1.09 [1.00–1.19]	1.05 [0.97–1.14]	1.06 [0.98–1.14]
Other	0.64 [0.52–0.78]	0.65 [0.53–0.81]	0.67 [0.53–0.82]
Missing	0.90 [0.82–0.98]	0.88 [0.82–0.96]	0.89 [0.82–0.97]
**Preferred Language**			
English	[REF]	[REF]	[REF]
Spanish	1.09 [1.01–1.18]	1.04 [0.97–1.11]	1.04 [0.97–1.11]
Other	0.65 [0.54–0.79]	0.66 [0.54–0.80]	0.66 [0.55–0.80]
Missing	0.70 [0.56–0.87]	0.71 [0.56–0.88]	0.71 [0.57–0.89]
**Payer**			
Commercial	[REF]	[REF]	[REF]
Medicaid	2.44 [2.26–2.64]	2.30 [2.14–2.47]	2.31 [2.15–2.48]
Medicare	1.73 [1.56–1.91]	1.64 [1.48–1.82]	1.64 [1.47–1.82]
Missing	2.22 [1.97–2.51]	2.14 [1.90–2.40]	2.14 [1.90–2.41]

IQR = interquartile range, SDoH = social determinants of health.

In the ADI model, those identifying as Non-Hispanic White had significantly lower odds of reporting HRSNs than those identifying as Non-Hispanic Black (OR 0.83, 95% CI 0.70–0.98), and those identifying as Hispanic had 9% greater odds of reporting HRSNs than those identifying as Non-Hispanic Black (OR 1.09, 95% CI 1.00–1.19). Aside from the above, race and ethnicity variables were not significantly associated with HRSN presence among the other area-level indices. In the ADI model, Spanish language preference was found to be significantly associated with greater odds of HRSNs. Compared with individuals with commercial insurance, individuals with Medicaid, Medicare, or missing insurance coverage information all had greater odds of reporting HRSNs across all area-level indices.

### Mapping

Figure 2a–c maps the overlapping prevalence of *individual-level HRSNs* and area-level SDoH measures among census tracts in the Bronx, NY. Separate maps are shown for the ADI score (Fig. 2a), SDI (Fig. 2b), and SVI score (Fig. 2c) for comparison in this descriptive geospatial analysis. Census tracts with both low prevalence of *individual-level HRSNs* (<19.3%) and low area-level SDoH scores are represented in gray. Census tracts with a high prevalence of *individual-level HRSNs* (≥19.3%) and low area-level SDoH scores are represented in light blue. Census tracts with a low prevalence of *individual-level HRSNs* (<19.3%) and high area-level SDoH scores are represented in pink. Lastly, census tracts with both high prevalence of *individual-level HRSN* (≥19.3%) and high area-level SDoH scores are represented in dark blue. Census tracts with fewer than 10 individuals screened for social needs are represented in white.

In comparing these three maps, we see that the ADI estimates a lower level of area-level SDoH for many of the census tracts with a high prevalence of *individual-level HRSNs* than the SDI and SVI, resulting in a greater proportion of light-blue than dark-blue census tracts in the ADI map. The SDI and SVI maps are similar in their estimation of area-level SDoH among the census tracts in the Bronx. However, in all three maps, we still see many census tracts with a high prevalence of *individual-level HRSNs* and low area-level SDoH (light blue).

**Figure 3.**
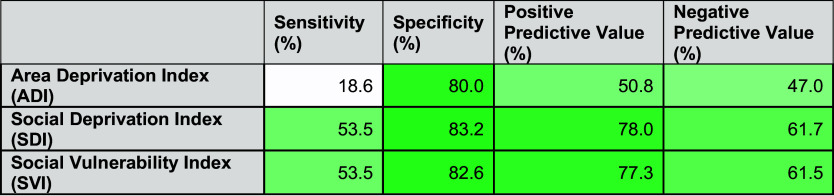
Sensitivity, specificity, and positive and negative predictive value of area-level social determinants of health indices.

Figure 3 displays the sensitivity, specificity, positive predictive values, and negative predictive values for each of the three area-level SDoH measures. In comparing these three indices, the ADI has a much lower sensitivity (18.6%), positive predictive value (50.8), and negative predictive value (47.0) compared to the other measures. The SDI and SVI are similar in their estimation of area-level SDoH among census tracts in the Bronx, with a greater specificity than sensitivity.

## Discussion

In this study, we examined the relationship between individual-level HRSNs routinely collected from health system patients and three separate measures of area-level SDoH (ADI, SDI, and SVI) within one historically marginalized urban county. In bivariate and multivariate analyses, the SDI and SVI indices both showed similar trends in predictive value to our individual-level HRSN data, with a greater likelihood of identifying individual HRSNs in communities with higher SDoH scores. However, while the specificity and positive predictive values of both the SDI and SVI were strong (∼83% and ∼78%, respectively), they had poor sensitivity and negative predictive value (∼54% and 62%, respectively), highlighting the challenge of relying on area-level indices alone in census tracts with lower SDoH scores. Scores from the ADI metric less predictably identified census tracts with higher HRSNs than either the SDI or SVI.

These findings are further emphasized in our mapping of the overlapping prevalence of area-level SDoH and individual-level HRSNs. This analysis is unique in its visualization of the distribution of these three commonly used area-level SDoH scores across one urban county. Expectedly, across all three maps, we see a concentration of both high *area-level SDoH* as well as high *individual-level HRSN* prevalence within neighborhoods in the Bronx that have historically experienced racial segregation via redlining [45]. However, census tracts with high rates of individual HRSNs can be seen distributed across the county. While the SDI and SVI are better able to identify these high-risk tracts than the ADI, there are significant gaps in their predictive ability that make the utility of these measures alone insufficient. Using these area-level measures as proxies may enable one to appropriately identify many individuals in the high SDoH risk communities but would miss many more in census tracts that are better resourced (i.e., lower area-level SDoH scores). Taken together, these data show that the SDI and SVI metrics function similarly to one another, and in distinct ways from the ADI metric. However, although more useful than the ADI, the SDI and SVI still miss much of the story of the individual experience of SDoH, which is not always clustered by census tract or geographic community. While we show here how these data are related but do not directly overlap with each other, further studies are warranted to understand the complementary ways these data can be used in the clinical environment and to inform advocacy at the community level.

This analysis adds to the growing body of literature comparing area-level SDoH metrics with individually measured HRSNs [28–33,46], cautioning against making assumptions about individuals using aggregate area-level data (also known as the ecological fallacy). Given these findings, there would be real risk in intervening within high-risk census tracts alone, as this would miss many of the individuals living in census tracts considered to have low SDoH risk scores but have self-reported HRSNs. Similarly, with many individuals in high-risk neighborhoods not reporting any HRSNs, the potential stigmatizing impact of designing programs based on the assumption of need for all individuals in those neighborhoods should not be ignored.

Others have come to similar conclusions over the past few years with slightly different methodologies or patient populations. Beckett et al. used Medicare claims and administrative data to create social risk factor “groups” (based on socioeconomic status, disability status, and race and ethnicity) that were used as proxies for individuals to compare with one area-level SDoH index (ADI) [28]. They concluded that neighborhood-level characteristics account for much less variation in social risk measures than individual-level HRSNs. Cottrell et al. linked census tract SDI scores with patient-level social risk screening data from a national network of community health centers and found that 40% of patients with at least 1 HRSN lived within neighborhoods classified as not disadvantaged [31], a similar finding to our 47% for SDI and SVI. However, the SDI metric identified 57% of individuals with no HRSNs living in disadvantaged communities in the Cottrell study, which varies considerably from the 17% in our study sample (corresponding to a specificity of 83%). A recently published follow-up study from the same network of community health centers expanded this analysis to include two additional area-level SDoH indices (the ADI and Material Community Deprivation Index) and quantified the relationships between these metrics and individual-level social risks [29]. They found that these area-level measures had low sensitivity and would likely miss most individuals with social risks, which is similar to our analysis, with the ADI metric missing ∼ 81% of individuals with HRSNs. In another study, Brown et al. explored how three area-level SDoH measures (the ADI, SDI, and Neighborhood Stress score) corresponded with survey results from a Medicare Advantage national sample assessing HRSNs and found similar discordance as our study between area-level SDoH measures and individual-level HRSNs [30].

Our results also varied from other studies using different methodologies. Ramphul et al. mapped individual food insecurity screening data from one health system in relation to one area-level SDoH index (the SVI) [33]. They found that census tracts with high SVI scores overlapped well with census tracts with high individual food insecurity, and census tracts with low SVI scores overlapped well with low individual food insecurity, with minimal outliers. Focusing on one individual HRSN (food insecurity) could potentially explain this variation in the findings from ours and others’ results. Miller-Rosales compared results from individual HRSN screening via five separate categories of HRSNs among patients during Medicaid enrollment with one area-level SDoH index (the Neighborhood Deprivation Index) [32]. Similar to our findings with SDI and SVI, they found that patients living in more vulnerable neighborhoods were more likely to report HRSNs, although this only applied to food insecurity and transportation barriers, and not financial stress, housing insecurity, or functional limitations. The magnitude of the effect size that they noted was also much lower than ours, with patients living in the most vulnerable neighborhoods having 1.07 greater odds of reporting any HRSN, as compared to a 1.80 greater odds (SDI) and 1.74 greater odds (SVI) in our sample. While these differences could be due to the different area-level metrics being used, the ways that HRSNs are screened for, and aggregated (i.e. individual needs [33], 5 categories [32], or 10 categories in this study) could explain some of this variation as well.

This study has important limitations that should be considered when interpreting its findings. First, the three area-level SDoH measures used here have slightly different variables built into their composite scores, which do not align directly with the domains measured in the individual HRSN screening tool. Importantly, the SVI measure includes variables of race and ethnicity, which we know are proxies for many of the other socioeconomic indicators that the tool measures and would likely be collinear with these, in addition to the race and ethnicity variables in the regression model. However, we have tested for and found no multicollinearity in the regression models. The timeframe of data collection was also different between the area-level measures and the individual HRSN screening data. Given that neighborhood demographics and economic circumstances likely change over time, we should be cautious in comparing these area-level indices as this may have contributed to some degree of variability in their association with individual HRSN. As HRSN screening becomes the standard of care across health systems, aligning the timeframes of these measures and analyzing them longitudinally may become easier and prove a fruitful avenue of investigation. Screening for HRSNs has also been implemented in a pragmatic fashion within this clinical setting [38], which has the potential to introduce a sampling bias for those patients screened for HRSNs. However, the demographics of those patients screened for HRSN match those of the health system as a whole, increasing our confidence in the representativeness of the sample (supplemental table E). Further research disaggregating some of these area-level measures and comparing them to individual HRSN data in a prospective, time-matched fashion could provide further insight into these relationships.

Despite these limitations, this study adds value in a number of substantive ways. First, the HRSN screening data was collected through routine visits at primary care sites throughout the ambulatory care network of an urban hospital system. This data collection methodology is likely more pragmatic than utilizing survey data not collected at the point of care, as was done in other similar studies [30,32]. Particularly given the new regulatory requirements, pragmatic approaches are better aligned with many health-systems efforts and quality measures to implement universal HRSN screening [19,47,48]. Additionally, the focus on one urban county in this analysis adds an important juxtaposition to analyses of health centers dispersed across national networks of health centers [29,31]. Finally, utilizing maps as a means of highlighting the heterogeneity of these findings we believe adds an important visual context for potential city and state policy implications.

The Bronx is often referred to in relation to historical divestment and marginalization [49] leading to poor health outcomes on the population level [50]. However, these data add important texture to this narrative with empiric evidence of resiliency in the face of structural violence, as shown with patients screening negative for HRSN despite living in high-risk and poorly resourced communities. While the accuracy of HRSNs screening can at times be limited by perceived stigma and social desirability when completing this screening questionnaire, we believe the sensitivity given to this screening initiative, in addition to the robust sample size we have analyzed, mitigates this potential limitation. Similarly, while community-level safety-net resource allocation is often determined by population metrics such as the indices in this analysis, we show evidence of many individuals struggling in communities around the Bronx despite the perception of low risk in those census tracts. Whether this is due to limited safety-net resources being allocated in those neighborhoods, or other factors we were unable to measure in this analysis, the heterogeneity of the experience of social risk and the distribution of structural determinants of health is put in stark relief in this analysis.

## Conclusions

We show here that within census tracts with the highest SDoH scores in one urban county, the SDI and SVI metrics are an adequate but imperfect proxy measure for predicting individual HRSNs. However, within census tracts with lower SDoH scores, the value of the SDI and SVI metrics is much more limited, not far from a coin flip in predicting individual HRSNs. As area-level SDoH scores continue to be developed and utilized in conjunction with risk adjustment within healthcare delivery [20,21], more studies are needed to understand the relationship between these area-level risk measures, their variability within different communities, and how they differ in comparison to individual HRSNs. While the granularity of measuring HRSNs is important, and clearly distinct from community-level risks, HRSNs can be transient and speak to individual problems. Area-level SDoH, however, can speak to systemic problems that require community interventions. Using these different measures together may strengthen an advocacy platform for resource allocation across different communities, both within health systems and within local and state governments. While more advocacy is needed for increased universal patient-level HRSN screening across health systems, leveraging both types of data to design targeted interventions is key for the multi-sectoral partnerships necessary to mitigate these risks.

## Supporting information

Telzak et al. supplementary material Telzak et al. supplementary material

## Supplementary material

The supplementary material for this article can be found at https://doi.org/10.1017/cts.2024.519.
