# Supplementary material for: Understanding individual health-related social needs in the context of area-level social determinants of health: The case for granularity
Source: J Clin Transl Sci. 2024 Apr 16;8(1):e78. doi: 10.1017/cts.2024.519 (PMC11091925; doi:10.1017/cts.2024.519)
Supplement: Telzak et al. supplementary material [file S2059866124005193sup001.docx]

**Supplemental Tables and Figures**

| **Supplemental Table A: Social Needs Screening Questions** | |
| --- | --- |
| **Social Need Screening Question** | **Variable name** |
| Are you worried that the place you are living now is making you sick? (Has mold, bugs/rodents, water leaks, not enough heat) | Housing Quality |
| Are you worried that in the next 2 months, you may not have a safe or stable place to live? (Eviction, being kicked out, homelessness) | Housing Instability |
| In the last 12 months, did you worry that your food could run out before you got money to buy more? | Food Insecurity |
| In the last 12 months, has lack of transportation kept you from medical appointments or getting your medications? | Health-related Transportation |
| In the last 12 months, did you have to skip buying medications or going to doctor’s appointments to save money? | Healthcare Costs |
| In the last 12 months, has the electric, gas, oil, or water company threatened to shut off services to your home? | Utility Costs |
| Are you finding it hard to get along with a partner, spouse, or family members? | Domestic Disputes |
| Do you need help getting childcare or care for an elderly or sick adult? | Child or Adult Care |
| Do you need legal help? (child/family services, immigration, housing discrimination, domestic issues, etc)? | Legal Help |
| Does anyone in your life hurt you, threaten you, frighten you or make you feel unsafe? | Interpersonal Violence |

| **Supplemental Table B: Comparison of Area-level Social Determinants of Health Metrics** | | | | |
| --- | --- | --- | --- | --- |
| **Domain** | **Item** | **ADI** | **SDI** | **SVI** |
| Income | Below Poverty Level | X | X | X |
|  | Below 150% of Poverty Level | X |  |  |
|  | Income Disparity | X |  |  |
|  | Median Family Income | X |  |  |
|  | Per Capita Income |  |  | X |
| Employment | Unemployment | X | X | X |
|  | White Collar Occupation | X |  |  |
| Education | High School Diploma or Higher | X |  |  |
|  | < High School Diploma |  | X | X |
|  | <9 years Education | X |  |  |
| Housing | Owner-occupied Housing | X |  |  |
|  | Rented Housing Unit |  | X |  |
|  | Median Monthly Mortgage | X |  |  |
|  | Median Gross Rent | X |  |  |
|  | Median Home Value | X |  |  |
| Household | Single-parent Households | X | X | X |
| Characteristics | Age 65+ Years |  |  | X |
|  | Age < 17 Years |  |  | X |
|  | Persons with Disability |  |  | X |
|  | Households Without a Telephone | X |  |  |
|  | Households Without a Motor Vehicle | X | X | X |
|  | Housing Without Complete Plumbing | X |  |  |
| Housing Type | Multi-unit (10+) Structures |  |  | X |
|  | Crowding (>1 person/room) | X | X | X |
|  | Mobile Homes |  |  | X |
|  | Persons in Group Quarters |  |  | X |
| Minority Status and Language | Non-hispanic White |  |  | X |
|  | Speak English ‘Less Than Well’ |  |  | X |


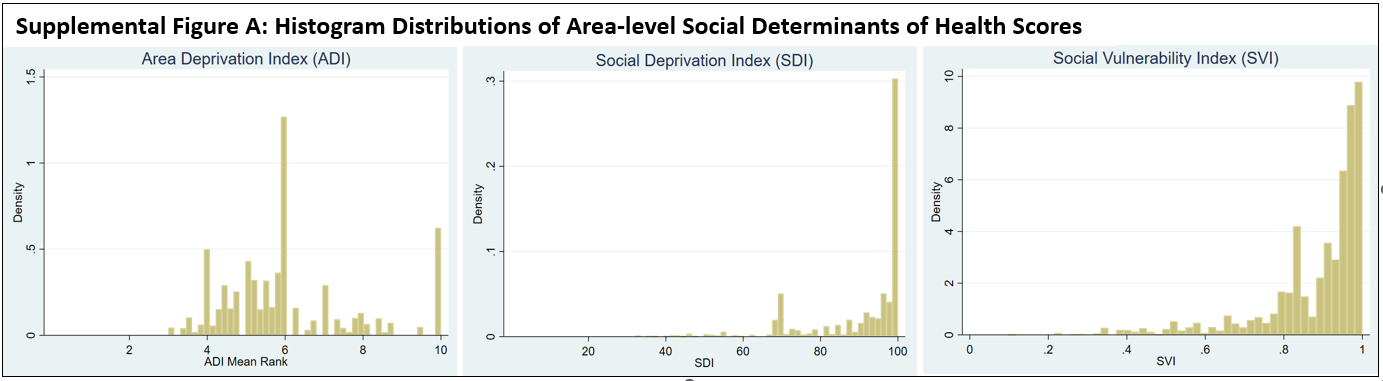


| **Supplemental Table C: Distribution of Reported HRSNs , N (%)*** | |
| --- | --- |
|  | |
| Housing Quality | 2,662 (5.9) |
| Money for Food | 2,611 (5.8) |
| Healthcare Transportation | 2,087 (4.6) |
| Housing Situation | 2,029 (4.5) |
| Healthcare Cost | 1,752 (3.9) |
| Domestic Disputes | 1,516 (3.4) |
| Utility Shutoff Threat | 1,372 (3.0) |
| Legal Help | 1,262 (2.8) |
| Child or Adult Care | 1,329 (2.9) |
| Interpersonal Violence | 548 (1.2) |
| *total N=8,396 |  |

| **Supplemental Table D: Count of Census Tracts Within Each Category of Area and Individual-level Risk** | | | | |
| --- | --- | --- | --- | --- |
|  | Low Area SDoH Score and Low Individual Need (Gray census tracts, N) | High Area SDoH Score and Low  Individual Need (Pink Census Tracts, N) | Low Area SDoH Score and High Individual Need (Light Blue Census Tracts, N) | High Area SDoH Score and High Individual Need (Dark Blue Census Tracts, N) |
| Map with Area Deprivation Index **(**ADI) | 124 | 31 | 140 | 32 |
| Map with Social Vulnerability Index (SDI) | 129 | 26 | 80 | 92 |
| Map with Social Vulnerability Index (SVI) | 128 | 27 | 80 | 92 |

| **Supplemental Table E: Comparison of Patients Screened for HRSN and General Medical Center Population** | | |
| --- | --- | --- |
|  | **Total Screened, N (%)** | **General Population (N, %*)** |
| **Total** | 45,279 | 308,687 |
| **Age (median [IQR])** | 33.5 [11.4-58.8] | 39 [16-60] |
| **Sex**  Female  Male  Transgender Non-Conforming | 27,018 (59.7)  18,261 (40.3) | 190,225 (61.6)  118,213 (38.3)  249 (0.0008) |
| **Race and ethnicity**  Non-Hispanic Black  Non-Hispanic White  Hispanic  Other  Missing | 12,737 (28.1)  1,518 (3.4)  17,866 (39.5)  1,005 (2.2)  12,153 (26.8) | 89,052 (28.8)  18,047 (5.8)  110,243 (35.7)  26,720 (8.7)  64,625 (20.9) |
| **Preferred Language**  English  Spanish  Other  Missing | 35,995 (79.5)  7,157 (15.8)  1,139 (2.5)  989 (2.2) | 250,384 (81.1)  46,486 (15.1)  8,863 (2.9)  2,955 (1.0) |
| **Payer**  Commercial  Medicaid  Medicare  Uninsured | 14,110 (31.2)  20,438 (45.1)  8,357 (18.5)  2,374 (5.2) | 105,035 (34.0)  138,698 (44.9)  61,069 (19.8)  3,885 (1.3) |

* unpublished health system data from 2021
